# Metabolic responses to sulfur dioxide in grapevine (*Vitis vinifera* L.): photosynthetic tissues and berries

**DOI:** 10.3389/fpls.2015.00060

**Published:** 2015-02-20

**Authors:** Michael J. Considine, Christine H. Foyer

**Affiliations:** ^1^School of Plant Biology, and The UWA Institute of Agriculture, University of Western Australia, Crawley, WA, Australia; ^2^Centre for Plant Sciences, School of Biology, Faculty of Biological Sciences, University of Leeds, Leeds, Yorkshire, UK; ^3^Irrigated Agriculture and Diversification, Department of Agriculture and Food Western Australia, South Perth, WA, Australia

**Keywords:** fruit, oxidative stress, antioxidants, quality, glutathione, SO_2_, elemental sulfur, wine

## Abstract

Research on sulfur metabolism in plants has historically been undertaken within the context of industrial pollution. Resolution of the problem of sulfur pollution has led to sulfur deficiency in many soils. Key questions remain concerning how different plant organs deal with reactive and potentially toxic sulfur metabolites. In this review, we discuss sulfur dioxide/sulfite assimilation in grape berries in relation to gene expression and quality traits, features that remain significant to the food industry. We consider the intrinsic metabolism of sulfite and its consequences for fruit biology and postharvest physiology, comparing the different responses in fruit and leaves. We also highlight inconsistencies in what is considered the “ambient” environmental or industrial exposures to SO_2_. We discuss these findings in relation to the persistent threat to the table grape industry that intergovernmental agencies will revoke the industry’s exemption to the worldwide ban on the use of SO_2_ for preservation of fresh foods. Transcriptome profiling studies on fruit suggest that added value may accrue from effects of SO_2_ fumigation on the expression of genes encoding components involved in processes that underpin traits related to customer satisfaction, particularly in table grapes, where SO_2_ fumigation may extend for several months.

## INTRODUCTION

Sulfur dioxide may be considered to be the “elephant in the room” of grape and wine industries and in agriculture more broadly, for both its health and environmental consequences. Or perhaps it is something of a “golden goose?” SO_2_ is used in >99% of wine production. About 15% of more than 15,000 patents for biological study of SO_2_ are related to wine (Chemical Abstracts Service, 2014). Therefore, grape berries and wine are an appropriate case for study and discussion of the metabolic responses of plant tissues and organs to SO_2_ exposure, particularly considering the responses of non-photosynthetic tissues to sulfur dioxide. Sulfur dioxide is considered here more as a food additive than an atmospheric pollutant and potentially phytotoxic agent. More specifically, we compare and contrast effects of SO_2_ in grape berries, which are reproductive organs, with the more expansive knowledge of the consequences of SO_2_ fumigation in leaves but we do not consider wine *per se*, which bears additional consequences beyond the life of the plant cell. In the following analysis, we do not distinguish between effects of SO_2_ on table and wine grapes. However, it is important to acknowledge the difference in the SO_2_ fumigation strategies that are applied in each case. Table grapes may be exposed to SO_2_ fumigation for several weeks for conservation. In contrast, the application for wine grapes is often only a few hours, prior to fermentation.

The grape and wine industries place high value on the knowledge and control of reductive and oxidative (redox) processes, and of microbial populations. Sulfur is capable of a wide range of oxidation states (–2 to +6), and hence sulfur-derived compounds are a major feature of redox metabolism and post-translational modifications, as well as defense and detoxification of toxins or heavy metals. Thiols and sulfides are among the most important flavor and aroma compounds and precursors in many wine varieties and styles, both desirable and undesirable (Baumes, 2009). Sulfur-derived compounds also play a major role in the abundance and stability of other flavor, aroma and texture components, such as tannins, phenolic acids, anthocyanins, and aldehydes.

**FIGURE 1:**
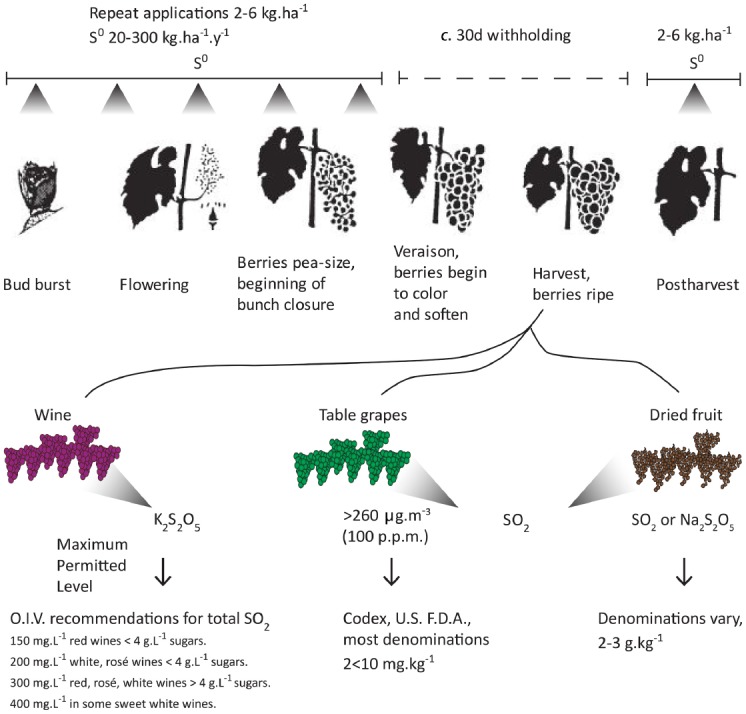
**Representative inputs of elemental sulfur (S^0^) and SO_2_, including sulfite salts, in grape and wine production.** Elemental sulfur is typically sprayed on vines prior to bud burst at rates of 2–6 kg.ha^–1^.y^–1^, repeated throughout the season. Application rates vary greatly, according to climate, wine style, fungal risk, and commercial preference. Many denominations mandate a > 30d withholding period for S^0^ application, largely due to adverse effects of residual sulfur on wine flavor and aroma qualities. For winemaking, sulfite salts (e.g., K_2_S_2_O_5_) or SO_2_ are added during the grape crush prior to fermentation for wine, as well as throughout fermentation and often at bottling to control microbial growth and for chemical antioxidant properties. For table grapes, SO_2_ is commonly used in storage shed, whether by forced fumigation in the cold room or by controlled release SO_2_-generating pads, particularly when fruit are intended for export or to be stored more than 1 week. Typically >260 μg.m^–3^ SO_2_ (100 p.p.m.) is required to surface-sterilize effectively, and thereafter a maintenance of 5–8 μg.m^–3^ (2–3 p.p.m.) has a fungistatic effect. The use in dried fruit prevents oxidative browning but is shown here only for additional context. It should be noted that the stated maximum residue levels are often far in excess of the actual amounts used in industry and detected in food surveys, e.g., sultana 2 mg.kg^–1^, 1000-fold below Maximum Permitted Level (MPL; F.S.A.N.Z., 2012). O.I.V., International Organization of Vine and Wine (www.oiv.it); Codex, Codex Alimentarius Commission (2014) (www.codexalimentarius.net/); U.S. F.D.A., U.S. Food and Drug Administration (2014) (www.fda.gov).

Sulfur is added to grapes and wine at several stages in several chemical forms, exploiting various chemical or toxicological properties (Figure 1). By mass, the greatest input is elemental sulfur (S^0^), which has some fungicidal activity (Williams and Cooper, 2004) but is largely used as a slow-release substrate for SO_3_^2–^, or SO_2_ when burned, which have fungicidal and fungistatic activities against most economically important pathogens, particularly powdery mildew (*Uncinula* spp.) and botrytis (*Botrytis cinerea* Pers. Fr.). It is also effective in control of mites, which can decimate bud vitality, damaging reproductive structures even before bud burst, and spiders for disinfestation of fruit postharvest. The abundance of elemental sulfur applied in most vineyards precludes any risk of sulfur deficiency (Robinson, 1988). Yet there is a large knowledge-gap in the speciation, chemical and metabolic, of sulfur-derived compounds between application in the vineyard and fermentation in the winery. The same is true of SO_2_ fumigation of fresh table grapes. To date there are no effective alternatives to S^0^ or SO_2_ application to control microbial infection, as grapes are highly susceptible to pathogens of different trophic habits; biotrophs, hemi-biotrophs and necrotrophs, and S^0^/SO_2_ are at least partly effective in controlling all, while being extremely cost-effective when used in combination with other agents such as copper. So while society and intergovernmental agencies maintain the threat of a complete ban, the grape industries would not survive, and typically act with great responsibility to minimize use within the Generally Recognized as Safe (G.R.A.S.) limits.

It is widely known that residual sulfur on berries promotes formation of off-flavors such as H_2_S during fermentation (Rankine, 1963; Kwasniewski et al., 2014). SO_2_ residues in fresh table grapes and wines are restricted by legislation to limit risks of ill-health effects in consumers. A few recent studies also reveal ecological pathways of applied sulfur in the vineyard (Hinckley et al., 2011; Hinckley and Matson, 2011). Each of these ignore the reality that atmospheric sulfur (e.g., SO_2_, H_2_S) is readily assimilated by plants (Rennenberg, 1984).

We recently demonstrated an expansive transcriptional reprogramming evoked by SO_2_ application to table grape berries (Giraud et al., 2012). The exposure was non-phytotoxic and at levels far below those acceptable in production, even in the context of organic grapes and wine. Here we explore the metabolic and quality consequences of such a large transcriptional footprint in the context of other studies that have documented pathways of elemental sulfur applied to grapevine, including retention in the berries. Importantly, we draw contrast to the broader literature on the consequences of SO_2_ exposure in leaves. While the impact of sulfur and sulfur-derived compounds in wine extends far beyond the strictly metabolic, or living activity of the grapes, this review is largely confined to that scope, but with hypotheses for the consequences for wine stability and sensory qualities. The chemistry of SO_2_ and sulfites in wine is adequately described in text books (e.g., Boulton et al., 1999). The influence of sulfites on fermentation and microbial activities is also beyond this review, as are the consequences of the many additional forms of sulfur inputs in the vineyard, including polysulfides (lime sulfur) and various thiols. However, we do consider elemental sulfur, as its application assumes oxidation to the antimicrobial oxide anions.

## SO_2_ CONCENTRATIONS: ISSUES AND INCONSISTENCIES

There is a notable inconsistency concerning the units of SO_2_ concentration used in the literature, particularly with regard to what constitutes a high or low concentration. This has led to difficulties in relating information in different studies. In fact, there are very few examples of the use of S.I. units of kg.m^–3^, or whether the volumes used refer to either a liquid or gas. This is not helped by the fact that the agronomic preference is to express units as parts per million (p.p.m.). Other researchers and various food industries use either mass- or volume per volume, e.g., μg.L^–1^ vs μL.L^–1^, or per mass, however, these units are not equivalent in terms of the amount of SO_2_ exposed to the plant or food. The information contained in this review largely concerns atmospheric concentration. We have therefore cited information in the S.I. units, referring to the density of 2.62 kg.m^-3^ SO_2_ at 25°C, 101.3 kPa (C.R.C., 2014), i.e., the volume-base unit is *c*. 382x the mass-base unit, hence if a particular study equates 1 p.p.m. to 1 μL.L^–1^, i.e., v/v, that is actually 2.62 μg.m^–3^ when expressed in S.I. units, i.e., 2.62 p.p.b.

An additional consideration when interpreting the results of different studies or contexts for atmospheric sulfur assimilation is the differences in the levels of flux of SO_2_ penetrating the tissues (Rennenberg and Herschbach, 2014). The numbers of stomata and their functional operation to control conductance is a major control point for SO_2_ influx into metabolism. When comparing leaves and fruit, it is important to point out that stomatal density is comparatively low in fruit, typically < 10 stomata per berry in grapevine (Palliotti and Cartechini, 2001). Moreover, the stomata on the berries are at least partly blocked with epicuticular wax (Rogiers et al., 2004). Postharvest storage conditions also maintain very low vapor pressure deficits, with high levels of relative humidity and low temperatures. Hence the capacity for influx of atmospheric sulfur would be manifold lower for fruit than leaves, especially when the considerable differences in surface area to volume ratios are taken into account.

A further inconsistency exists among the data from the grape, food and wine industries, where SO_2_ application is reported per unit volume, which may be gas or liquid, while residues are reported per unit mass or liquid. For example, postharvest application of SO_2_ to table grapes is based on units per volume of gas, while residues are based on units per mass of extracted berry [e.g., maximum permitted level 30 mg.kg^–1^ Codex Alimentarius Commission (2014), 10 mg.kg^–1^ U.S. Food and Drug Administration (2014), refer Figure 1]. Hence, p.p.m. application of SO_2_ does not directly relate to p.p.m. residues. We do not attempt to resolve this inconsistency here but are careful to distinguish the two.

It is pertinent to also provide a more environmental context to understand the range and trends in global SO_2_ emissions and atmospheric concentrations in industrial and natural environments. Global SO_2_ emissions have declined >15% since 1990, although only peaked in China *c*. 2006 and emissions in India were still increasing in 2010 (Klimont et al., 2013). From 1980 to 2013 atmospheric SO_2_ surveys in the United States of America showed a mean atmospheric SO_2_ declined from >400 μg.m^–3^ to < 80 μg.m^–3^ (E.P.A., 2014). In 2011 > 96% of Chinese cities were 20–150 μg.m^–3^, which is within their grade I and II air quality guidelines, which refers to protected conservation environments, and rural and residential areas, respectively (State Environmental Protection Administration, 2013). The World Health Organization air quality guidelines are 20 μg.m^–3^ (24 h mean) or 500 μg.m^–3^ (10 min mean; W.H.O., 2006). Internationally, air quality standards vary, e.g., >350 μg.m^-3^ (24 h mean) for Bangladesh, Indonesia, and Singapore (Clean Air Initiative, 2010). In addition, some denominations use 24- or 1-h means, while the WHO is committed to a 10-min mean (W.H.O., 2006).

## SULFUR ASSIMILATION AND SEQUESTRATION IN LEAVES

The preservative effects of SO_2_ have been exploited in winemaking since antiquity. Despite this, the post-industrial contexts of ecological and agricultural damage have attracted far more scientific enquiry on the mechanisms and consequences of SO_2_ exposure to plants. In recent decades, pollution-prevention measures have decreased such threats, and acute SO_2_ injury is now much less common. Sulfur deficiency can be experienced by plants in the natural environment, leading to changes in plant morphology, metabolism and gene expression (Honsel et al., 2012). In particular, levels of the antioxidant thiol metabolite, glutathione are decreased leading to a de-repression of sulfate uptake and assimilation (Hartmann et al., 2004). Similarly, it is not uncommon for field crops to suffer from low level sulfur deficiency, resulting in changes in nitrogen metabolism and leading to the accumulation of amino acids such as asparagine (Shewry et al., 1983). For example, asparagine accumulates to very high levels in wheat is grown under conditions of sulfur deficiency. This is important because sulfur availability is the most important factor affecting the acrylamide-forming potential of wheat grain (Muttucumaru et al., 2006).

The highly regulated processes of sulfur uptake, assimilation and distribution throughout the plant have been extensively reviewed (refer to Takahashi et al., 2011; Koprivova and Kopriva, 2014, and references therein). Sulfur uptake is considered to be driven by the demand for core sulfur-containing compounds, such as cysteine and glutathione (Davidian and Kopriva, 2010). Sulfur depletion initially leads to an increase in sulfate uptake from the soil, while further limitation results in redistribution, driven by sink capacity. The multiple tiers of transcriptional to hormonal and metabolic regulation, including by sugars, triggered by sulfur depletion illustrate the vast importance of sulfur metabolism to plants.

Low sulfur-dependent restrictions on glutathione accumulation in plants (Nikiforova et al., 2003) are likely to limit the stress tolerance, because of the multiple roles of this abundant non-protein thiol, particularly in secondary metabolism and oxidative signaling (Noctor et al., 2012). *Arabidopsis* mutants lacking high affinity sulfate transporter, SULTR1:2, have decreased levels of glutathione (Maruyama-Nakashita et al., 2003). Moreover, overexpression of genes encoding sulfur-assimilation enzymes SERINE ACETYLTRANSFERASE (SAT) and *O*-ACETYLSERINE(THIOL)LYASE (OASTL) increased cysteine and glutathione contents in *Arabidopsis*, potato and tobacco leaves (Harms et al., 2000; Noji and Saito, 2002; Wirtz and Hell, 2007).

High atmospheric SO_2_ concentrations can have both positive and negative effects on plant growth and development (Gayler and Sykes, 1985). Plants can rapidly assimilate SO_2_ and H_2_S into reduced sulfur pools and sulfates, leading to improved growth especially in soils with poor sulfur availability. An atmospheric level of ≥ 30 nL.L^-1^ SO_2_ (79 ng.m^–3^) can contribute 10-40% of leaf sulfur assimilation (De Kok et al., 2007; Zhao et al., 2008). Elevated SO_2_ concentrations around natural CO_2_ springs can lead to an enhanced accumulation of sulfur metabolites and proteins in surrounding vegetation (Rennenberg, 1984; Schulte et al., 2002). However, these effects vary greatly between species (Naito et al., 1994), as SO_2_ exposures as low as 2-5 nL.L^-1^ (5-13 ng.m^-3^) can cause reductions in growth (Heber and Huve, 1998), and even visible injury to leaves and other vegetative tissues. High SO_2_ levels can lead to visible injury in young leaves is with chlorosis and necrotic inter-vein areas in broad-leaved species, and chlorotic spots and brown tips in pine conifers (Rennenberg, 1984). This is often caused by an accumulation of sulfite and sulfate and associated with very high leaf sulfur contents. SO_2_ gas enters leaves via stomata and at apoplastic pH is hydrated and oxidized successively to sulfite and sulfate, both of which can inhibit photosynthesis and energy metabolism if they accumulate. The SO_2_-induced inhibition of photosynthesis and associated increase in the oxidation state of leaf cells underpins the toxicity syndrome.

Within a normal physiological range (3–76 S g.kg^–1^ FW; Zhao et al., 2008), sulfate assimilation leads to the synthesis of L-cysteine, which is the precursor for the synthesis of a range of sulfur-containing metabolites such as methionine and glutathione. The two final reactions in this sequence are catalyzed by a cysteine synthase complex, which is comprised of two enzymes SAT and OASTL. SAT is responsible for the acetylation of L-serine by acetyl-CoA to produce *O*-acetylserine (OAS). OASTL catalyzes the formation of cysteine from H_2_S and OAS. The SAT family consists of five members in *Arabidopsis*, three of which are localized to the cytosol, one in chloroplast stroma, and one in mitochondria (Kawashima et al., 2005). The mitochondrial and chloroplast SAT forms make the major contribution to cysteine synthesis under optimal and stress conditions (Haas et al., 2008; Watanabe et al., 2008). The SAT protein is unstable when not associated with OASTL, and hence SAT activity in the chloroplasts and mitochondria is regulated by the assembly and maintenance of the cysteine synthase complex. The chloroplast SAT form (SAT1), which is considered to be the rate-limiting enzyme in cysteine biosynthesis in leaves interacts with cyclophilin CYP20–3, which located in the chloroplast stroma. CYP20–3 foldase activity is influenced by thioredoxin-mediated reduction and is considered to link photosynthetic electron transport activity and oxidative regulation to the folding of SAT1, and hence SAT1 activity and cysteine biosynthesis (Dominguez-Solis et al., 2008). Thus, SO_2_-mediated oxidation of the chloroplast stroma might directly influence the flux and capacity of cysteine synthesis.

In addition to the reductive pathway of sulfur assimilation described above, which is localized in chloroplasts, there is also an oxidative pathway for the removal of sulfite derived from SO_2_ that is localized in the peroxisomes, in which SULFITE OXIDASE (SO) plays a predominate role. While the significance of the SO pathway relative to the reductive pathway in the chloroplasts remains to be established for example in terms of relative flux (Rennenberg and Herschbach, 2014), SO is considered to be important in the maintenance of intracellular sulfate pools, and to contribute to metabolic recycling and potentially act as a sink pathway for excessive sulfur (Hänsch et al., 2007; Brychkova et al., 2013).

Leaves exposed to non-phytotoxic levels of SO_2_ (600 nL.L^–1^; 1.6 ng.m^–3^) show a wide range of transcriptome, metabolic and enzymatic changes in *Arabidopsis*, indicating a large scale reprogramming at both transcriptional and translational/post-translational levels. SO_2_ (sulfite) enters the plastid sulfur assimilation pathway downstream of sulfate, immediately downstream of the major rate-limiting enzyme ADENOSINE 5′-PHOSPHOSULFATE REDUCTASE (APR), and upstream of SULFITE REDUCTASE (SIR) and OASTL/SAT. In general, enzyme activities upstream of sulfite were repressed, including APR, although its transcription was unaffected (Hamisch et al., 2012; Randewig et al., 2012). This indicated a repression of further sulfite synthesis, while sulfate accumulated. However, metabolism of sulfite was enhanced, via increased SIR and SAT activities, effecting increased cysteine and glutathione contents, although again, transcriptional regulation was more marginal. Transcripts encoding proteins involved in nitric oxide synthesis and antioxidant defenses as well as apoplastic peroxidases and defensins were also upregulated (Hamisch et al., 2012). These transcriptional signatures were very similar to those seen in *Arabidopsis* leaves exposed to much higher concentrations (30 μg.L^–1^; 30 mg.m^–3^; Zhao and Yi, 2014), which was phytotoxic and reduced the growth rate, but not lethal (Li et al., 2008). Insight can also be drawn from SO knock-out mutants in *Arabidopsis*, which showed even more marked transcript and activity reductions in APR when exposed to SO_2_, indicating strong downregulation of sulfite synthesis, while cysteine, glutathione and thiols were markedly increased (Hamisch et al., 2012; Randewig et al., 2012).

There are a number of similarities between the responses of leaf metabolism to SO_2_ and to the metabolic production of hydrogen peroxide (H_2_O_2_). For example, photorespiration-induced oxidative stress in *Arabidopsis* mutants deficient in CATALASE (*cat2*) led to extensive glutathione accumulation and triggered increases in transcripts encoding APR and SAT (Queval and Noctor, 2007). The chloroplast SAT is strongly induced by H_2_O_2_ and by glutathione (Queval and Noctor, 2007). Moreover, oxidation triggers post-translational activation of γ-GLUTAMYL CYSTEINE SYNTHETASE (γ-ECS) and APR, possibly by oxidation-triggered decreases in the reduced glutathione (GSH): glutathione disulfide (GSSG) ratio that may allow glutaredoxin (GRX)-mediated activation of both enzymes (Noctor et al., 2012). The H_2_O_2_-induced increases in glutathione accumulation in catalase-deficient barley mutants were accompanied by increased uptake of labeled sulfate (Smith et al., 1985). Similarly, the large increases in glutathione accumulation achieved in transgenic plants with ectopic expression of a bacterial enzyme having both γ-ECS and GLUTATHIONE SYNTHETASE activities were dependent on having a high sulfur supply (Liedschulte et al., 2010).

Taken together, these observations suggest that enhanced cellular oxidation is a hallmark of SO_2_ action in leaves. However, SO_2_-induced changes in cellular redox state are important in facilitating enhanced rates of sulfur assimilation, oxidative activation being a trigger for both cysteine and glutathione synthesis. Presumably, SO_2_-induced damage only occurs when the oxidative activation of these pathways fails to restore the cellular redox balance. Major differences in the effects of SO_2_ observed between vegetative and reproductive tissues may therefore be attributed to the presence or absence of photosynthesis, with its inherent sensitivity to oxidative inhibition and the relative metabolic activities of different types of plastids. In addition, variations in the barriers to gas exchange and the surface area to volume ratios may lead to differences in SO_2_ sensitivity between vegetative tissues such as leaves and reproductive organs such as fruit.

## SULFUR IN THE VINEYARD, WINERY AND PACKING SHED

**FIGURE 2:**
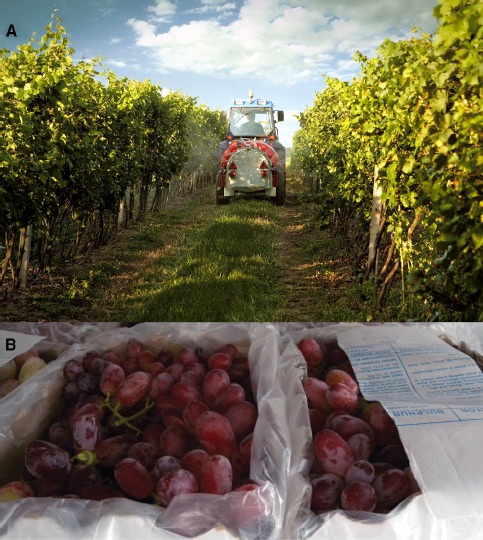
**Application of elemental sulfur (S^0^), as wettable sulfur (A) in the vineyard during the growing season, as well as postharvest application of SO_2_ to table grapes from SO_2_-generating pads (B).** Applications of wettable sulfur, as well as other sulfurous pesticides vary greatly across industry and climatic zones (Figure 1). Unless destined for immediate sale, table grapes are treated with SO_2_, typically with the use of SO_2_-generating pads such as the one seen on top of berries in the right hand side box of **(B)**. The fruit **(B)** had been stored at 2–4°C for 4 weeks, with (right) or without (left) SO_2_, showing no visible quality differences. Panel **(A)** rights purchased from ShutterStock (www.shutterstock.com).

Elemental sulfur (S^0^) is widely and frequently applied during the growing season, typically in the form of wettable powders, sprayed directly on vines to provide a “protective” coating, or alternatively burned in the vineyard. Both assume oxidation to SO_2_/SO_3_^2–^/HSO_3_^–^, which are effective in controlling the pathogen, albeit with differing efficacies. The reported range of wettable S^0^ used in commercial vineyards, including certified organic vineyards, varies by several orders of magnitude (Figures 1 and 2A). For example, agrochemical companies in Australia typically recommend up to 100 kg.ha^-1^.yr^-1^, and while many wineries may use as little as 20 kg.ha^–1^.yr^–1^, reports internationally, where pathogen pressures are higher, vary up to 600 kg.ha^–1^.yr^–1^ (Hinckley and Matson, 2011). Recent studies have shown that much of the S^0^ may be oxidized within minutes or hours and is ultimately lost from the vineyard via hydrological pathways (Hinckley et al., 2011; Hinckley and Matson, 2011). A significant pool of S^0^ was retained in the soil surface, and likely the vegetative surface, until irrigation or rain events. Within 7–12 days, the initial surge in topsoil (0–0.5 m) sulfates had declined to pre-application levels. Using the dynamic changes in sulfur species in above- and below-ground fractions, and scaling to vineyard-scale, the authors concluded that any accumulation of sulfur in the soil and plant matter was lost during rain events in the dormant season. Yet, *c*. 2% (w/w) of applied sulfur was retained in the berries, which in the context of biomass represented 7-14 kg.ha^-1^.yr^-1^.

Although ecologically revealing, the above study isn’t greatly informative for the biologist, grower, winemaker or consumer, as the study was dynamic, with no control *per se*, and the forms of sulfur on or within the berries could not be discriminated. Earlier studies in wheat using ^35^S^0^ showed rapid assimilation of up to 2% (w/w) of applied sulfur into sulfate, glutathione and amino acids (Legris-Delaporte et al., 1987). The most prominent concern to winemakers is that S^0^ residues may result in increased H_2_S production during fermentation (Rankine, 1963; Kwasniewski et al., 2014). For this reason most practices require a >30 day withholding period (Figure 1), which typically results in S^0^ residues of < 1 mg.kg^–1^ (Kwasniewski et al., 2014). At this level, the effect on H_2_S production is thought to be negligible, particularly for white wines, where settling and limited skin contact, result in near-complete removal of S^0^ before fermentation. However, before and during the crushing process, SO_2_ or K_2_S_2_O_5_ is added at levels to give appropriate levels of free SO_2_ (20-50 g.m^–3^ in liquid). The metabolic impacts of those additions are difficult to dissect from chemical effects and beyond this review.

## SULFUR DIOXIDE ASSIMILATION AND METABOLISM IN THE BERRY

The prolonged or repeated postharvest applications of SO_2_ to fresh table grapes have been a mainstay of the table grape industry for decades. The maximum residue level for fresh table grapes is 10 p.p.m. (10 mg.kg^–1^). Between the 1920’s and 1980’s, before the US Food and Drug Administration suspended sulfiting agents from the register of GRAS additives, an initial fumigation of 13–26 mg.m^–3^ (5–10,000 p.p.m. in air) was common, followed by repeat fumigations of 6.5 mg.m^–3^ at 7–10 day intervals (Nelson and Baker, 1963). Although this practice is still widely used in some regions, e.g., California, USA (Crisosto and Smilanick, 2014; Luvisi, 2014), international practice is far more conservative and increasingly sophisticated through the use of SO_2_-generating pads, particularly where fruit are to be exported (Figure 2B). Such pads are impregnated with Na_2_S_2_O_5_ in a polymeric matrix that, upon hydration enables a transient burst of >260 μg.m^–3^ (100 p.p.m. in air), which is sufficient to surface-sterilize, followed by sustained release of 5-8 μg.m^–3^ (2–3 p.p.m. in air) for several weeks to prevent re-infection (Clemes, 1986; Palou et al., 2002).

Only a limited number of studies have rigorously investigated the absorption and oxidation of SO_2_ in the berry. Peiser and Yang (1985) used a combination of radiolabeled and unlabeled SO_2_, and carefully managed extraction technique to control oxidation, to calculate that *c*. 10% (w/w) of the applied SO_2_ rapidly accumulated in the berry as sulfites (4-5 mg.kg^–1^ berry FW), in free and bound forms (hydroxyl sulfonic acids of aldehydes and methyl- and cyclic-ketones). The authors found *c*. 70% of the absorbed sulfites were rapidly oxidized to sulfate with a half-life of 4 h, with most of the remainder oxidizing with a half-life of 20 h. A more recent study with similar technique showed similar rate of uptake but more sustained retention of sulfites of ≥ 30% (Lagunas-Solar et al., 1992). Both studies concluded that inorganic sulfur formed the major pool of retained sulfur, with little or no evidence of assimilation to organic forms, such as thiol amino acids, proteins and sulfolipids. If so, this would contrast greatly with foliar assimilation, suggesting major differences in the metabolic activity of the plastids. To date in grape berries, only the ultrastructure of plastids have been presented (Fougere-Rifot et al., 1995), however, a recent study of the bioenergetics of tomato chromoplasts demonstrated significant functional rearrangement of electron transport (Renato et al., 2014), which may suggest that sulfur assimilation is also altered.

**FIGURE 3:**
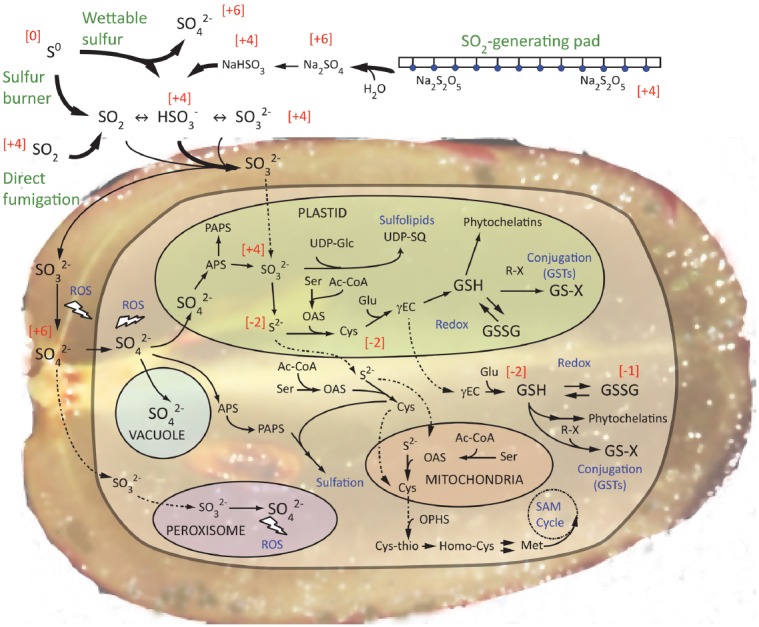
**Outline of the pathways of sulfur assimilation in the grape berry from major viticultural inputs of sulfur (S^0^), SO_2_ and sulfites.** Atmospheric SO_2_ and sulfites, shown here generated from elemental sulfur (wettable or burned), SO_2_ fumigation or SO_2_-generating pads, will hydrate predominantly to bisulfite (HSO_3_^–^) at apoplastic and subcellular pH ranges [apoplast pH5-6 (Grignon and Sentenac, 1991), cytosol, plastid stroma and nucleus *c*. pH7.2, peroxisome and mitochondria *c*. pH >8 (Shen et al., 2013) and vacuole *c*. pH3.5 (Fontes et al., 2012)]. However, for simplicity, we’ve only shown SO_3_^2–^ within the cell. Arrow thickness and relative font size of metabolites represents hypothesized downregulation of sulfite synthesis and oxidation, and accumulation of cysteine, glutathione and related metabolites. Dotted lines represent unconfirmed transport steps. Numbers in square brackets represent oxidation states of the sulfur atom. ROS, reactive oxygen species; APS, adenosine 5′-phosphosulfate; PAPS 3′-phosphoadenosine 5′-phosphosulfate; OASï-acetyl serine; Ser, serine; Ac-CoA, acetyl co-enzyme A; UDP-Glc; UDP-glucose; UDP-SQ, UDP-sulfoquinovose; Cys, cysteine, γEC, γ-glutamylcysteine; GSH, glutathione (reduced); GSSG, glutathione disulfide (oxidized); R-X, substrate electrophile (e.g., xenobiotic, flavonoid); GS-X, glutathionylated substrate (by GSTs, GLUTATHIONE *S*-TRANSFERASES); OPHS, *O*-phosphohomoserine; Cys-thio, cystathionine; Homo-cys, homocysteine; Met, methionine; SAM, *S*-adenosylmethionine. Adapted from Giraud et al. (2012) with permission.

Previously, we have shown that substantial reprogramming of the grape berry transcriptome occurs after 21 days of fumigation with a commercial SO_2_-generating pad, which generated up to 260 μg.m^–3^ within 8 h of application, declining to 26 μg.m^–3^ by 24 h and sustaining 3–8 μg.m^–3^ for at least 8 weeks (Figure 2B; Giraud et al., 2012). The number of SO_2_-responsive transcripts, both up- and down-regulated was several-fold larger and different from the sole or combined effects of salicylic acid or methyl jasmonate, which are both well-known elicitors of plant transcriptional response. The net transcriptome signature of sulfur assimilation suggested that oxidation to sulfate in the apoplast and peroxisome had reached a saturation point, and that sulfite was directed toward alternative paths, including conjugation, and sulfation. The data suggest that sulfur was also directed toward cysteine, methionine and particularly glutathione (Figure 3), as has been observed in *Arabidopsis* leaves, albeit to a limited extent (Van der Kooij et al., 1997; De Kok and Tausz, 2001). Glutathione and enzyme activities associated with glutathione metabolism, including GLUTATHIONE-S-TRANSFERASE (GST) and other thiols play important roles in plant responses and acclimation to a range of abiotic and biotic stresses. In SO_2_-treated *Arabidopsis* leaves, water-soluble thiol accumulation comprised only 2% (w/w) of the assimilated sulfur (Van der Kooij et al., 1997), however, the berry differs in several ways, not least because sulfur cannot be mobilized to other organs. Previous studies have shown that “super-nutritional” levels of sulfur can enhance the innate defenses of plants and crops (Bloem et al., 2005; Kruse et al., 2007; Nakamura et al., 2009). Our transcript data showed up-regulation of several orthologs of *GST*, however, the microarray format was not completely representative of the sulfur metabolic pathways, for example lacking an ortholog of *GLUTATHIONE SYNTHETASE* (Giraud et al., 2012). That study was also limited in metabolic data, which we are currently investigating along lines of thiol metabolism.

The broader picture suggests that SO_2_-fumigation may have a number of value-adding effects on the quality of the berry. In an earlier study, with comparable treatment, we’d shown that total phenolic acids and *in vitro* total antioxidant capacity were increased in SO_2_-treated berries (Considine et al., 2009). Notwithstanding our reservations of *in vitro* assays of total antioxidants (Mubarak et al., 2012), the transcriptome signature suggested that anthocyanin synthesis was enhanced, as well as a number of other processes that may contribute to improved retention of berry quality postharvest, particularly preservation of texture and flavor qualities.

## THE ADDED VALUES OF SO_2_-FUMIGATION

Sugars, organic and amino acids, and soluble pectins are the major soluble solids in grapes. The fruit soluble solids concentration (SSC%, °Brix) and titratable acidity, together with texture are the major determinants of the fruit taste and quality. Postharvest practices implemented by the industry, however, focus on the weakest link, being infection, loss of turgor and cell wall degradation, rather than flavor, even though the taste and flavor of table grapes are key components of marketability. The transcriptome data suggest that SO_2_-fumigation may have the potential to improve traits such as sugar profiles and soluble pectin content. For example, transcripts encoding grapevine orthologs of PECTIN METHYLESTERASE and PECTATE LYASE, as well as GALACTINOL SYNTHASE, which is the first committed step in synthesis of raffinose family oligosaccharides, were increased in grape berries after SO_2_-fumigation (Giraud et al., 2012). These transcripts have previously been shown to be highest late in the ripening stages of grape berries (Guillaumie et al., 2011). However, enhanced activities of these enzymes could also lead to softening of the berry and accumulation of raffinose oligosaccharides, which would tend to have a negative impact on grape quality. Nevertheless, it may be possible to maximize the effects of SO_2_ fumigation to improve the outcome of current practices leading to long-term to enhanced postharvest soluble solids contents.

In relation to the wine industry, SO_2_ serves several purposes postharvest, including limiting oxidation and controlling microbial populations at least until the inoculated yeast can dominate fermentation. As glutathione and thiols are widely known to be important determinants of wine sensory attributes, a major contribution of postharvest SO_2_ is to maintain their stability. It is unknown to what extent the SO_2_ may augment their levels through assimilation in the berry, whether from S^0^ or SO_2_, or through yeast assimilation.

While more in-depth studies are required to determine whether the observed changes in pectin and sugar metabolism-related transcripts are translated into effects on sugar composition, the possibility remains that such changes could result in alterations in soluble solids contents and hence improve berry quality. It would be worthwhile to explore this possibility, together with more comprehensive studies on the effects of SO_2_ on the content and composition of secondary metabolites. For example, there is little evidence to date that anthocyanin synthesis is changed as a result of SO_2_ fumigation (Giraud et al., 2012) although the lower abundance of flavan-3-ol transcripts after SO_2_ fumigation suggest that anthocyanins are not degraded as rapidly. Further studies are required to explore such possibilities, together with the effects of the duration of SO_2_ exposure on quality-linked traits such as tannin contents in wine and table grapes.

## CONCLUSION

Evidence suggests that there are not only differences in the susceptibility of different plant species to SO_2_, but variations in the effects of SO_2_ on the different organs of the same plant. While gaps in current knowledge remain concerning the mechanisms that prevent SO_2_-induced damage in some tissues but not others, the marked contrast in the metabolic consequences of SO_2_-exposure in photosynthetic and non-photosynthetic tissues suggests that in the absence of photosynthesis plant organs are highly tolerant to SO_2_. The available transcriptome and metabolic data from leaves and fruit demonstrate that in both vegetative and reproductive organs atmospheric SO_2_ is preferentially metabolized to SO_4_^2–^. The high requirement of SO_2_ metabolism for cellular reductants results in an increases in cellular oxidation. The resultant shift in cellular redox state that provokes much of the broader transcriptional reprogramming that is observed in leaves and berries, in an attempt to restore the cellular redox balance. At levels that are currently used in grapevine industries, SO_2_ appears to have beneficial effects on quality, and more importantly to industry, does not appear to be damaging, or to compromise quality. In contrast, leaves have a much lower threshold of sensitivity that is orders of magnitude lower than the fruit, in terms of the potential to induce damage. This differential sensitivity is not just due to variations in the physical structure of the two tissue types in terms of the barriers to diffusion but also to functional organization of the plastid, particularly the operation of the photosynthetic electron transport chain in the thylakoid membrane together with the highly redox-sensitive enzymes of carbon assimilation and associated metabolism. However, such factors might not form the basis for a large distinction between table and wine grapes, in which there is photosynthetic activity at least during the first growth phase because of the limitations imposed by limited stomatal numbers and conduction on SO_2_ penetration into the photosynthetic cells.

## AUTHOR CONTRIBUTIONS

MC and CF co-wrote the manuscript.

### Conflict of Interest Statement

The authors declare that the research was conducted in the absence of any commercial or financial relationships that could be construed as a potential conflict of interest.
